# Prevalence and co-occurrence of addictive behaviors among former alternative high school youth

**DOI:** 10.1556/JBA.3.2014.005

**Published:** 2014-02-03

**Authors:** Steve Sussman, Thalida Em Arpawong, Ping Sun, Jennifer Tsai, Louise A. Rohrbach, Donna Spruijt-Metz

**Affiliations:** Institute for Health Promotion and Disease Prevention Research, University of Southern California Keck School of Medicine, Alhambra, CA, USA

**Keywords:** multiple addictions, prevalence, co-occurrence, latent class analysis, addiction groups, convergent validity

## Abstract

*Background and Aims:* Recent work has studied multiple addictions using a matrix measure, which taps multiple addictions through single responses for each type. *Methods:* The present study investigated use of a matrix measure approach among former alternative high school youth (average age = 19.8 years) at risk for addictions. Lifetime and last 30-day prevalence of one or more of 11 addictions reviewed in other work (Sussman, Lisha & Griffiths, 2011) was the primary focus (i.e., cigarettes, alcohol, other/hard drugs, eating, gambling, Internet, shopping, love, sex, exercise, and work). Also, the co-occurrence of two or more of these 11 addictive behaviors was investigated. Finally, the latent class structure of these addictions, and their associations with other measures, was examined. *Results:* We found that ever and last 30-day prevalence of one or more of these addictions was 79.2% and 61.5%, respectively. Ever and last 30-day co-occurrence of two or more of these addictions was 61.5% and 37.7%, respectively. Latent Class Analysis suggested two groups: a generally Non-addicted Group (67.2% of the sample) and a “Work Hard, Play Hard”-addicted Group that was particularly invested in addiction to love, sex, exercise, the Internet, and work. Supplementary analyses suggested that the single-response type self-reports may be measuring the addictions they intend to measure. *Discussion and Conclusions:* We suggest implications of these results for future studies and the development of prevention and treatment programs, though much more validation research is needed on the use of this type of measure.

## Introduction

A variety of behaviors have come to be considered addictions by researchers and practitioners (Demetrovics & Griffiths, 2012), delineated by common features (e.g., preoccupation, loss of control) and, in fact, the First International Conference on Behavioral Addictions took place in Budapest, Hungary in March, 2013 which demonstrated research consensus on the existence of multiple types of addictions (see: http://icba.mat.org.hu/; accessed April 25, 2013). Substance addictions pertain to excessive intake of substances such as drugs or food, whereas behavioral (process) addictions pertain to engaging in behaviors (e.g., work, shopping, or sex) addictively (Sussman et al., 2011). Some studies have been completed to try to ascertain (a) the prevalence of substance and behavioral addictions and (b) the co-occurrence of two or more addictions, to better understand the extent to which addictions are more a problem of person (i.e., a statistically vulnerable minority) or of lifestyle (i.e., among many people, except those who are relatively resilient). For example, Sussman et al. (2011) examined data from 83 studies with sample sizes of at least 500, supplemented by smaller scale studies, to address these questions pertaining to 11 addictive behaviors over a 12-month period. The addictions examined were to cigarettes, alcohol, other/hard drugs, eating, gambling, Internet, shopping, love, sex, exercise, or work. They found that the 12-month prevalence of these 11 addictions among U.S. adults averaged 47% of the population, with a 23% co-occurrence (of two or more addictions). They suggested that addictions are just as likely to be a problem of modern, sedentary lifestyles as of neurobiological vulnerability.

For two main reasons, few studies have examined multiple addictions in youth utilizing extensive measures of each addiction. First, assessment through use of multiple inventories takes a great deal of time, which may not be practical particularly in large youth survey samples. In such samples (usually in school settings, but also in mailed or telephone-delivered versions), researchers generally are granted only 50 minutes to administer a survey (Sussman, Dent, Stacy, Burton & Flay, 1995). Thus, only a few addictions can be measured at the same time. Second, there is a great deal of redundancy in the measurement of various addictions, which may share in common such features as involving appetitive motives (e.g., pleasure, arousal or sedation, nurturance), brief periods of satiation, preoccupation, loss of control, and accumulation of a variety of negative life consequences (Sussman & Sussman, 2011). Such redundancy is burdensome to measure. Thus, several previous studies have examined multiple addictions as a matrix measure. With this type of self-report measure, several addictions are tapped, generally with one item per type of addiction, arranged in a matrix format. While an addiction matrix measure does not extensively measure any addiction, and validation studies of such measures have not been conducted, this approach is practical, economical, and may actually tap different addictive behaviors.

Cook (1987) was the first researcher to investigate use of a matrix measure to identify prevalence and co-occurrence of addictive behaviors. In a sample of 604 U.S. college students, he examined 10 among the 11 focal addictive behaviors (i.e., cigarettes, alcohol, illicit drugs, eating disorders [obesity, anorexia, and bulimia], gambling, shopping, relationships/love, sex, exercise [running], and work), along with additional addictions (e.g., caffeine), violence, and emotional disturbance constructs. He did not examine Internet addiction, due to the year the study was completed (i.e., the Internet as we know it today did not exist at the time). The highest prevalence addictions reported were: relationships/love (25.9%), caffeine (20.1%), work (17.5%), sex (16.8%), shopping (10.7%), alcohol (10.5%), and cigarettes (9.6%). He found that approximately a quarter of the sample (23.8%) responded “no” to all addictive behaviors, violence, or emotional disturbances, suggesting that a high prevalence of addictive behaviors exists. However, it must be noted that he did not separate between addictive behaviors, partner violence, and emotional disturbances when reporting that statistic. In addition, after creating “logical clusters”, he found that all of the addictions were significantly associated with each other except for running/work/shopping with alcohol/illicit drugs. One might conjecture whether or not a contrast was being demonstrated between prosocial daily activity-type addictions versus risky drug use-related addictions.

Alexander and Schweighofer (1989), in a partial replication study of 136 Canadian college students, found similar prevalence findings as Cook (1987) on two of the addictions (relationships and work), but prevalence was much lower on other categories (based on how use was described [as addiction, negative addiction, dependence or regular use]). Defined only as regular use, prevalence was actually higher than the Cook sample on all types of addictions. Greenberg, Lewis and Dodd (1999), in a sample of 129 college students, found significant inter-correlations among nine addictions (alcohol, caffeine, chocolate, cigarettes, exercise, gambling, Internet use, television, and video games) except for exercise with alcohol and cigarette smoking, cigarette smoking with chocolate, and videogames with chocolate and exercise. Highest prevalence addictions were exercise (30%), caffeine (29%), television (26%), alcohol (26%), cigarettes (23%), and chocolate (23%), which were higher than Cook among the same addictions measured.

MacLaren and Best (2010), with a sample of 948 college students, examined the factor structure of a set of 16 addictions. Three factors were identified: (a) nurturant (e.g., compulsive helping [dominant and submissive], work, shopping, food [binging and starving], exercise, relationships [dominant and submissive]), (b) hedonistic (illegal drugs, alcohol, tobacco, and sex) factors, and (c) another hedonistic-like factor (prescription drugs, gambling, caffeine). Highest prevalence addictions were exercise (25.6%), shopping (21.8%), relationships dominant and submissive (17% and 11.9%), caffeine (16.5%), food starving and binging (16.4% and 14.9%), compulsive helping dominant and submissive (12.5% and 12.1%), work (12.4%), prescription drugs (12.2%), sex (10.3%), and alcohol (10.2%). Though not replicated by MacLaren and Best (2010), earlier work by this same research group also had delineated dominant and submissive factors nested within nurturant and hedonistic factors (Christo et al., 2003; Haylett, Stephenson & Lefever, 2004). Two of these studies were conducted with college undergraduate students, but Haylett et al. (2004) studied 543 consecutive admissions to the PROMIS Recovery Center (mean age = 35 years). Perhaps, additional factors emerge as a function of addiction severity or age of the sample studied.

The present study is the first to examine the use of a matrix addiction measure with former continuation high school youth. Alternative high school youth, in general, are not able to remain in mainstream education because of an inability to obtain graduation credits in a timely manner due to functional problems (e.g., absenteeism, drug use). “Continuation” high school is the name of the alternative school system in California (USA). Continuation high schools were created to fulfill a State mandate that all youth 16 years of age or older receive part-time education until they are 18 years of age (California Educational Code Section 48400; established in 1919), within the high school district in which they reside. These youth report a higher prevalence of tobacco and other drug use than same age peers from the regular (comprehensive) high school system and are likely to report a higher prevalence of other addictions as well (Sussman, Dent & Galaif, 1997).

In this study, we measured former continuation high school youth three years after participating in a drug abuse prevention project (see Sussman, Sun, Rohrbach & Spruijt-Metz, 2012). We focused on the 11 addictions identified by Sussman et al. (2011). We examined the prevalence of these 11 addictions (within a larger set of 22 addictions), using an addictions matrix measure. We also examined the prevalence of co-occurrence of two or more of these addictions among this population.

In addition, we utilized a person-centered latent variable approach to examine the underlying pattern of addictive behaviors to differentiate groups of youth. Latent Class Analysis (LCA) is a multivariate approach, which assumes that an underlying categorical latent variable determines one’s class membership and yields distinct profiles based on students’ responses to a set of items (Hagenaars & McCutcheon, 2002; Lazarsfeld, 1950; McCutcheon, 1987). One benefit of using LCA models is that statistical fit indices can be used to evaluate model fit and help decide on the number of classes that best fit the data, along with substantive considerations.

Finally, we investigated whether or not these single response items contained within an addiction matrix measure are associated with other measures of these addictive behaviors; this might suggest convergent validity for the use of the matrix measure. Specifically, we examined the associations of cigarette, alcohol, other/hard drug use, sex, Internet, and exercise addictions with other available measures from the questionnaire that measured these addictions in other ways.

## Methods

### Subjects

Subjects were 717 former continuation high school youth in southern California, who had attended any of 24 schools 3 years prior, as part of a drug abuse prevention program (Sussman et al., 2012). Participants averaged 19.8 years of age (*SD* = 0.8 years), 52.4% were male, 66.5% were Hispanic, 10.8% were non-Hispanic White, 22.7% were Other ethnicity, and approximately 64.9% reported that at least one parent completed high school.

### Data collection

Data were collected as a 3-year follow-up of a drug abuse prevention project (Sussman et al., 2012) through three methods: telephone, mailings from the office, and home visits (surveys administered at the home and completed immediately or mailed back to the office). First we attempted to call subjects. For those we reached by telephone, we either completed the survey by telephone or mailed surveys to the home if the subject preferred that method. If we were not able to reach subjects by telephone after multiple attempts, we mailed surveys to the subject’s home. We also attempted to reach subjects by traveling to the subject’s home. Some subjects completed surveys right away at the home; other subjects preferred holding on to the survey and mailing them back to us. Of the 717 surveys completed, 58% were completed by telephone, 16% were completed via home visits (half of those were completed immediately, half were mailed back in within two weeks of the visit), and 26% were returned through mailings sent to the home from the office.

### Measures

*Addictions.* The current study used a multi-response addiction matrix measure. This measure began with categories developed by Cook (1987), followed by feedback provided in pilot sessions with one class of alternative high school youth and two classes of college undergraduates. Subjects endorsed ever and past 30-day addiction categories that applied to them, and could write in additional addictions that they felt they experienced. The final version of the matrix measure included responses reported by at least 10 subjects in the pilot study. After completing the measure, they were asked for feedback regarding wording of the measure’s items to assist in enhancing its clarity.

The final measure header is: “Sometimes people have an addiction to a certain drug or other object or activity. An addiction occurs when people experience the following: they do something over and over again to try to feel good, for excitement, or to stop feeling bad; they can’t stop doing this thing, even if they wanted to; bad things happen to them or to people they care about because of what they are doing.” Next to the header subjects were asked: “Have you ever been addicted to the following things?” and “Do you feel you are addicted to them now (in the last 30 days)?” Twenty-two response categories of addictions were provided along with a 23rd which permitted participants to indicate an open-ended response to “Any other addiction? Please identify: ____”

The categories were: cigarette smoking; alcohol drinking; marijuana use; other drugs (such as cocaine, stimulants, hallucinogens, inhalants, XTC, opiates, valium or others); caffeine (coffee, or energy drinks such as Red Bull); eating (way too much food each day, binge eating); gambling; Internet browsing (surfing the web); Facebook, Myspace, twitter, MSN, YM, or other online social networking; texting (cell phone use); online or offline videogames (PS3, Xbox, Wii); online shopping; shopping at stores; love; sex; exercise; work; stealing; religion; self-mutilation (cutting, skin picking, hair pulling); driving a car; gossip; or any other addiction. For the purposes of the present study, only 11 categories were emphasized for most analyses, to approximate the categories examined in the Sussman et al. (2011) study. Marijuana was combined with the other drugs response category to reflect other/hard (illicit) drug addiction. Internet browsing and Facebook categories were combined to create an Internet addiction category. The online or offline video-games category was not included in the Internet addiction category because gaming might have been offline. Shopping at stores and online shopping were included to assess shopping addiction.

*Demographics.* Demographic information was collected on age (in years), gender, ethnicity (coded as Latino/Hispanic, White/Caucasian, or other [African American, American Indian/Native American, mixed or other], and parental educational status. Parent education was measured across both parents, derived from a 6-level variable ranging from “did not complete 8th grade” to “attended or completed graduate school”, and was coded as to whether at least one of the parents graduated high school or not.

*Compulsive Internet Use (CIU).* A 4-item index was used to assess problematic Internet use (Davis, Flett & Besser, 2002). The subset of items measuring diminished impulse control was employed for the current study; pertaining to how often problematic use happened. The items were “I use the Internet more than I ought to”, “I usually stay on the Internet longer than I had planned”, “Even though there are times when I would like to, I can’t cut down on my use of the Internet”, and “My use of the Internet sometimes seems beyond my control”. The Likert-type response options were (1) Never, (2) Rarely, (3) Sometimes, (4) Most of the time, and (5) Always. The CIU construct showed a good internal consistency (Cronbach’s alpha = 0.81). The mean of all 4 items was used as a continuous measure of CIU.

*Risky sexual behavior.* Participants were asked three items about risky sexual behavior which tapped frequency (as in Griffin, Botvin & Nichols, 2006; Sussman et al., 2012). They were asked two items pertaining to the “last 12 months” and “last 30 days”: “...with how many people have you had sexual intercourse?”. Responses were “0”, “1”, “2”, in increasing increments of 1 up to “more than 10 people” (11 response categories). They were also asked “In the past 30 days, how many times did you have sexual intercourse?” Responses were “0”, “1 to 5 times”, “6 to 10 times”, “11 to 15 times”, up to “more than 30 times” (eight response categories).

*Exercise.* Three fill-in-the blank exercise items were asked, one for each of “strenuous”, “moderate”, and “mild” exercise. For example, the strenuous exercise item read: “In the past 7 days, did you do strenuous exercise that made your heart beat fast for more than 15 minutes like running, biking, soccer, or carrying boxes or furniture?” Subjects indicated number of times in the past 7 days, as a fill-in-the-blank type item. These three items were adapted from the Godin Leisure-Time Exercise Questionnaire (GLTEQ; Godin & Shephard, 1985).

*Substance use.* Participants were asked, “How many times in the last month have you used…” each of various substance use categories (e.g., cigarettes, alcohol, drunk on alcohol, marijuana, cocaine, hallucinogens, etc.). Response options were provided to indicate 0 to over 100 times (1 = 0 times, 2 = 1–10 times, 3 = 11–20 times, …, 12 = over 100 times). The present study utilized four categories of drug use: cigarettes, alcohol, drunk on alcohol, and other drug use (marijuana, cocaine, hallucinogens, stimulants, inhalants, ecstasy, pain killers, tranquilizers, or other hard drugs; Cronbach’s alpha = .83), creating continuous scores for each (all log transformed). The reliability of the alcohol, tobacco and other drug-use (ATOD) item format used here has been previously established (e.g., Graham et al., 1984; Needle, McCubbin, Lorence & Hochhauser, 1983).

*Substance abuse.* An index of overall substance abuse was created using 4 questions (e.g., “In the last 12 months, have you kept using alcohol or drugs even though it was keeping you from meeting your responsibilities at work, school, or home?”), with yes-no binary responses, serving as proxy items of the DSM-IV substance abuse disorder categories. For this study, responses were summed into a single, continuous variable of substance abuse in the past year (Cronbach’s alpha = .66).

Self-reported problem consequences of drug use were established in the current study with use of the Problem Consequences Subscale of the Personal Experience Inventory (PEI-PCS; Sussman et al., 1997; Winters, Stinchfield & Henly, 1993). The measure assessed 11 personal consequences of substance abuse (e.g., “In the past 12 months, how many times have you sold personal things like your clothes or jewelry to get or pay for alcohol or other drugs?”) on 4-point scales (1 = none to 4 = often [10 or more times]). The PEI has been recommended by the National Institute on Drug Abuse (NIDA) for use in evaluating adolescent substance abuse (Winters et al., 1993). The Personal Consequences subscale provides good discriminant validity between interview-derived diagnostic groups (e.g., no diagnosis, abuse, dependence; point biserial correlation = .72). It is perhaps the best self-report measure available to assess adolescent substance abuse disorder because of its length (only 11 items), ability to tap content that is more than just drug use *per se,* and its relatively high prediction of involvement with drug treatment (Winters et al., 1993).

### Ethics

The study procedures were carried out in accordance with the Declaration of Helsinki. Subjects were informed that their participation was voluntary and that they could withdraw from participation at any time without penalty. Confidentiality of responses was emphasized for all subjects. Questionnaires were identified by number-only on computer. Subjects also were notified that a Certificate of Confidentiality had been achieved to legally protect responses provided. The Institutional Review Board of the University of Southern California-Health Science Campus approved the study and reviewed it annually. All subjects were informed about the study and all provided informed consent.

## Analysis and Results

We created the same 11 addiction categories as in the Sussman et al. (2011) review. Ever and last 30-day prevalence of one or more of these 11 addictions was 79.2% and 61.5%, respectively. Co-occurrence of two or more addictions, ever and last 30-days, was 61.5% and 37.7%, respectively. The average number of lifetime addictions was 2.48 (*SD* = 2.13) and the average number of addictions in the past 30 days was 1.48 (*SD* = 1.68). Expanding the number of categories to 22 addictions raised ever and last 30-day prevalence, and co-occurrence, to 84.8% and 68.2%, and 72.0% and 51.2%, respectively (slightly higher).

Ever (lifetime) addicted on the 11 addictions in order from highest prevalence to lowest prevalence was: love (34.3%), Internet (29.3%), other/hard drugs (29.2%), exercise (27.2%), cigarettes (24.3%), sex (24.1%), binge eating (23.4%), work (20.6%), shopping (17.9%), alcohol (14.8%), and gambling (3.2%). Last 30-day addiction in order from highest prevalence to lowest prevalence was: love (23.2%), Internet (18.4%), exercise (17.7%), sex (16.5%), cigarettes (13.4%), binge eating (12.7%), other/hard drugs (12.7%), work (15.6%), shopping (9.9%), alcohol (5.7%), and gambling (1.8%). The prevalence of ever addicted and last 30-day addiction showed a nearly identical pattern across addictions, except that other drug addiction was relatively less prevalent among the behaviors for 30-day addiction versus ever addicted.

All descriptive statistics and correlation coefficients were run in SAS Version 9.3 (SAS Institute Inc., 2012–2013). Chi-square comparisons were run for each of the 11 addiction categories, for both ever and last-30 day addiction, comparing general method of collection (telephone versus paper completion). Of 22 comparisons, only five were significant (*p* < .05). These were for alcohol (ever and last 30-day), sex (ever and last 30-day), and binge eating (last 30-day). In these cases, prevalence reports by telephone were lower than by paper questionnaire. While significant, the magnitudes of the differences were small (all comparisons less than 7%) for alcohol and binge eating, but larger for sex (13% for ever and for last 30-day).

### Latent Class Analysis of the 11 addictions

Latent Class Analysis (LCA) is a useful method for identifying homogeneous subgroups within a heterogeneous population with categorical data. LCA was conducted to determine addiction group categorization based on students’ responses to the 11 dichotomous (yes, no) last 30-day behaviors. Of primary interest were class probabilities (the probability that subjects belonged to a type of addiction group) and item probabilities within classes (the probability that subjects engaged in a type of addiction within an addiction group). Because LCA is an exploratory method, no assumptions were made about the structure or distribution of classes *a priori.* To conduct the analysis, a series of LCA models were iteratively constructed, starting with the most parsimonious one-class model and fitting successive models with an increasing number of latent classes. To determine best-model fit, a combination of statistical indicators was used. We evaluated the Pearson chi-square, likelihood ratio chi-square, Akaike Information Criterion (AIC; Akaike, 1987), Bayesian Information Criterion (BIC; Schwartz, 1987), Lo–Mendell–Rubin Likelihood Ratio test for mixture distributions (LMR; Lo, Mendell & Rubin, 2001), and entropy values. LCA models were tested using the MPlus Version 6.0 software program (Muthen & Muthen, 2004).

We failed to find a difference between Class 2 and Class 3 (*p* = .72), which suggested a two-class solution. This finding provides statistical differentiation between addicted and non-addicted subjects; that is, less than 10% of Class 1 subjects endorsed any of the 11 addictions (and less than 6% endorsed eight of them), whereas over 21% of Class 2 subjects endorsed each of the 11 addictions except for alcohol (14%) and gambling (4.3%). Additional fit indices were evaluated to determine whether the 2-class solution works maximally. The AIC suggested that it was the best-fitting model with an AIC for two-classes = 5628.154 and three-classes = 5616.992. Entropy was slightly lower for the two-class solution (65.8%) compared to the three-class solution (66.5%). Also, the differences in BIC scores between models were very small (BIC for two-classes = 5733.381; for three-classes = 5777.120).

**Figure 1.**
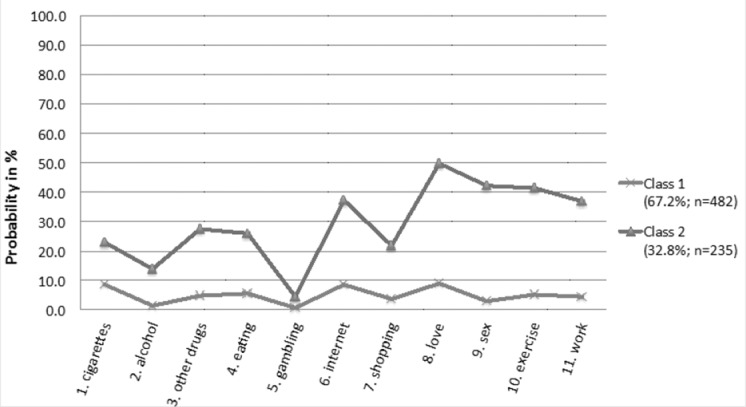
Latent class probabilities for endorsement of each addictive behavior

*Notes:* Vertical axis is the probability in percentages for each addiction.

Item-response probability values shown in Table 1 and Figure 1 indicated that the two-class solution provided substantive interpretability for contrasting addiction versus non-addiction groups (McCutcheon, 1987; Muthen & Muthen, 2004). We examined the latent class probabilities of endorsement of each addictive behavior. The members of Latent Class 1 (67.2% of the sample) reported being below 10% on all 11 addictions. They reported highest prevalence on love (9.1%), cigarettes (8.4%), and Internet (8.4%) addictions. They reported lowest prevalence on gambling (0.5%), alcohol (1.3%), and sex (2.8%) addictions. Because of the low prevalence of addictions overall, this might be labeled as the *Non-addicted Group* (in general).

**Table 1.** Results of Latent Class Analysis (LCA) retaining two classes

	Class 1 (67.2%; *n* = 482)	Class 2 (32.8%; *n* = 235)
1. Cigarettes	8.4	22.8
2. Alcohol	1.3	14.0
3. Other drugs	4.9	27.3
4. Eating	5.7	25.8
5. Gambling	0.5	4.3
6. Internet	8.4	37.3
7. Shopping	3.5	21.9
8. Love	9.1	49.7
9. Sex	2.8	42.2
10. Exercise	5.2	41.3
11. Work	4.3	37.0

*Notes:* The numerical values are percentages. These were calculated using Mplus (6.0).

The members of Latent Class 2 (32.8% of the sample) reported high general prevalence of addiction at over 21% for all items except for gambling (4.3%) and alcohol (14.0%). Highest prevalence addictions for this group were love (49.7%), sex (42.4%), exercise (41.3%), Internet (37.3), and work (37.0). Outside of gambling and alcohol, they reported lowest prevalence on shopping (21.9%), cigarettes (22.8%), and eating (25.8%) addictions. With the higher overall prevalence on all items, but particularly those that indicate prosocial behaviors, this group might be labeled as a *“Work Hard, Play Hard”-addicted Group*.

### Convergent validity analyses

For the next set of results, all *p*s < .0001, unless otherwise reported. Point biserial correlation coefficients were calculated, looking at the association of a continuously measured comparison measure with endorsement of an addiction matrix item. The associations of the last 30-day cigarette smoking item with self-reported ever and last-30 day addiction to cigarettes was .59 and .79, respectively. The associations of last 30-day alcohol use with self-reported ever and last-30 day addiction to alcohol was .21 and .36, respectively. The associations of last 30-day getting drunk on alcohol with self-reported ever and last-30 day addiction to alcohol was .29 and .45, respectively. The associations of last 30-day marijuana or other “hard” drug use with self-reported ever and last-30 day addiction to marijuana or other drug use was .41 and .55, respectively. Substance abuse disorder was associated with ever and current addiction to cigarettes (.25 and .23), alcohol (.30 and .33), and marijuana or other drug use (.31 and .34). The PEI-PCS was associated with ever and current addiction to cigarettes (.25 and .28), alcohol (.32 and .28), and marijuana or other drug use (.33 and .28). The associations of number of people with whom one has had sexual intercourse in the past 12 months, the number of people one has had sexual intercourse with in the past 30 days, and the number of times one had sexual intercourse in the past 30 days with ever being a sex addict was .24, .25, and .29. The associations of these same three items with being a sex addict over the past 30 days were .24, .33, and .35.

The associations of the Internet addiction index with the combined ever or last 30-day browsing and Facebook item was .41 and .49, respectively. The associations of the Internet addiction index with single addiction matrix items, considering all computer-related categories of ever being addicted to Internet browsing, Internet social networking, online or offline videogame playing, or online shopping was .45, .36, .13 (*p* = .0004), and .15, respectively. The associations of Internet addiction with past 30-day Internet addiction browsing, social networking, online or offline video gaming, or online shopping was .54, .41, .18, and .12 (*p* = .001), respectively.

Finally, the associations of how many times one engaged in strenuous exercise, moderate exercise, and mild exercise in the past 7 days with ever being addicted to exercise was .08 (*p* = .08), .01 (ns), and .01 (ns). The association of these three measures of exercise with exercise addiction in the past 30 days was .12 (*p* = .007), .04 (ns), and .01 (ns). Thus, only current engagement in strenuous exercise was significantly related to current exercise addiction.

## Discussion and Conclusions

The last 30-day prevalence of these 11 addictions in the present study is similar (within 5%) to the Sussman et al. (2011) 12-month adult prevalence data regarding cigarettes, alcohol, gambling, and shopping (work also only differed by 5.6%). In another recent 12-month prevalence study of Canadian adults (Konkoly Thege et al., 2013), the current results are similar (within 5%) on these same four addictions plus work. The former continuation high school youth reported much higher prevalence on other/hard drug use, Internet, and sex addictions, compared to both recent adult studies (Konkoly Thege et al., 2013; Sussman et al., 2011). Also, compared to the earlier Sussman and colleagues study, the current sample reported much higher prevalence on eating, love and exercise addictions. Konkoly Thege and colleagues did not measure love and exercise addictions. However, the former continuation high school youth reported lower prevalence of current eating addiction than in the Konkoly Thege study (which was approximately 20%). The relatively large difference between all three studies on eating addiction prevalence may be due to the way in which eating addiction was defined (e.g., as binge eating disorder by Sussman et al. [2011] versus eating too much or too little by Konkoly Thege et al. [2013]). The overall prevalence of one or more addictions was 10% higher among the current sample than the Konkoly Thege et al. (2013) study, and about 15% higher than the Sussman et al. (2011) study. This would make sense since this was a young at-risk sample.

The two-class LCA analysis solution was retained based on the overall pattern of the statistical indicators of class determination. The class structure in the current study did not differentiate among different types of addictions. Possibly, because this is an at-risk, young sample, and we did not look only at the subsample that reported one or more addictions, LCA supported a simple model. Alternatively, these results could support an argument that many of these addictions are exchangeable; one may even speculate that these 11 addictions may serve as potential substitute addictions for each other. As it does appear to be the case that addictions share common neurobiological underpinnings (e.g., mesolimbic dopaminergic turnover), perhaps a two-class solution would not be all that surprising (Sussman et al., 2011).

Furthermore, in the present study, the addicted group tended to participate in addictions involving generally legal, relatively prosocial activities that an emerging adult might engage in during one’s daily life (love, sex, exercise, Internet, and work). The substance addictions, cigarettes (22.8%), alcohol (14.0%), other drugs (27.3%), and eating (25.8%), were of far lower prevalence within this group. Thus, we labeled them the “Work hard, play hard” addicted group. This pattern of entrenchment in relatively conventional activity-type addiction is more the norm of addictive behavior (e.g., Cook, 1987; MacLaren & Best, 2010), even among the present sample of at-risk young people.

However, previous work does tend to differentiate among different types of addictions in samples of college youth and chemically dependent adults (e.g., Haylett et al., 2004; MacLaren and Best, 2010). Further, some previous work suggested dominance-submissive, pleasure, or nurturance appetitive motives (see Haylett et al., 2004; Sussman, 2012). It makes sense to think that youth may gravitate toward relatively conventional, nurturant (e.g., workaholism) versus extreme, hedonistic (e.g., hard drug use) addictions, depending on the life experiences, vulnerability, and the appetitive motives being sought (Sussman, 2012). An appetitive motives conception is consistent with the speculation that addictions are misdirected or excessive motives (instincts), and that different factors may reflect different general appetitive motives (Sussman, 2012). The present results could lead one to suggest that addictions are essentially guided or directed within lifestyle contexts (Csikszentmihalyi & Larson, 1984; Sussman, Stacy, Ames & Freedman, 1998), which do not obviously reflect distinct appetitive motives. A future replication study with the current type of sample is needed, as well as additional work with other populations, since only a few such addiction matrix-addiction class type studies have been completed.

Finally, cigarette, alcohol, other/hard drug, sex, Internet, and exercise addiction single items were associated significantly with other corresponding measures, suggesting convergent validity of these items with other addiction-related constructs. The matrix measure conceptualization appears to have some value, although additional studies with more lengthy inventories of addictions would be useful. Also, we did not have corresponding measures for five of the addictions (e.g., love, work).

### Limitations and future research

There are at least five limitations of the present study. First, differences in sampling could bias prevalence estimates, although the relative pattern of addiction prevalence and co-occurrence was similar comparing paper versus telephone-completed data. Also, the confidentiality of the protocol used would serve to minimize response bias. Still, one cannot rule out report biases due to sampling.

Second, while the addiction matrix-type measure has been investigated in some previous work, as described in the Introduction, much more work on the validation of addiction matrix-type items is needed. Also, too few studies exist to confirm the existence of stable addiction co-occurrence factors or latent groups. Arguably, this type of measure might be better termed as “self-perceived addiction” rather than as “addiction” though we maintained the same usage as in the previous studies.

A third limitation with the current study as with its predecessors is the lack of information on the deeper meanings of latent groups uncovered through LCA or factor analytic approaches. One has to infer what the groups likely represent. Some recent work has investigated the relations of types of addictions with personality factors (e.g., Andreassen et al., 2013). Possibly, this type of work may assist in identifying underlying meanings in these latent groups. Use of qualitative approaches (e.g., focus groups) may assist as well. Theoretically, as an example, one might think of these 11 addictions as grouping to reflect active-nurturance (e.g., Internet, shopping, work), active-pleasure seeking (e.g., sex, love, exercise), and passive-pleasure seeking (alcohol, cigarette, other drug use, eating) motives. Possibly, providing subjects with a list of appetitive motives or lifestyle contexts, and asking them to place types of addictions within each might be a way to approach the dimensionality of the addictions in a different way.

A fourth limitation is that while most of the point biserial correlation coefficients between other measures with addiction matrix items were significant, only 20 of 42 associations showed values of at least .30. Also, the measures used as comparisons may be subject to a variety of demand or other effects that surveys of large samples will tend to fail to address. Clinical interviews are an obvious, more sensitive means to examine the validity of these addiction matrix items. Still, this is the first such examination and, as such, is important.

Finally, these data were cross-sectional. We have no idea of the stability of different addictions. It is possible that some addictions (e.g., alcohol) are more immutable than others (e.g., work [one may lose their job] or exercise [one may get injured].) Longitudinal data are needed to discern this possibility. As of yet, there are no longitudinal studies that employ an addiction matrix-type measure.

Future studies might address shifting trends in addictions and the implications of being addicted to certain behaviors versus others. That is, self-reported prevalence on measures of addiction may change as the acceptability of being addicted to certain behaviors change, along with varying associations. For example, one might associate being addicted to love, sex, exercise, or work with social images including “romantic” or as examples of “modern living”. These addictions may be considered more acceptable than being addicted to cigarettes, alcohol, and/or other drugs, and the latter addictions may be associated with “rebellious” or “loss of self-control” types of social images. However, social images may be changing pertaining to some drugs; in particular marijuana use. Marijuana use may become an addiction of higher prevalence and associated with relatively positive images (e.g., “being modern”), over the next several years. Perhaps, marijuana addiction should be considered separately from other drug addiction matrix items in future longitudinal work. Changes in patterns of addiction over time may be important to explore in future work using an addiction matrix measure.

In summary, the present study contributed to a body of knowledge on prevalence, co-occurrence, latent class structure, and convergent validity of multiple addictions, using an addiction matrix measure, as applied to former continuation high school youth. As with previous studies, the present study highlights the high prevalence and co-occurrence of the addictions among youth and adults. Lifestyle context factors may drive a tendency toward addictions among people, and perhaps severity of addictions might reflect such variables as common neurobiology. Prevention and treatment programming may need additional resources to better meet the needs of assessment and tailoring of programming to different addictions, but perhaps a “generic” perspective of addiction might be applied across large populations given the results of the present study. Finally, it is possible that societal-level changes are needed to reduce modern lifestyle predictors of addictions (e.g., pressure to perform, breakdown of the extended family). We may speculate that much physical, social, and emotional negative consequences are resulting from engagement in these several types of addictions. Much future work is needed in this arena, as addiction is undoubtedly much more widespread than we care to admit.
